# Diabetes linked oral complications in south Indian cohorts

**DOI:** 10.6026/97320630019085

**Published:** 2023-01-31

**Authors:** Manisha K, Vishnu Priya Veeraraghavan, Kaviyarasi Renu, Kavitha Sankaran, Gayathri Rengasamy

**Affiliations:** 1 Department of Biochemistry, Saveetha Dental College and Hospitals, Saveetha Institute of Medical and Technical Sciences, Saveetha University, Chennai, Tamil Nadu, India, 600 077

**Keywords:** Hyperglycaemia, diabetes, oral complications, biochemical parameters

## Abstract

Diabetes is characterized by elevated blood sugar and insulin resistance. In poorly controlled or uncontrolled diabetes, persistent hyperglycemia causes oral and systemic problems. Therefore, it is of interest to evaluate biochemical indicators for oral health and diabetes using a fully automatic biochemistry analyzer which separates patient serum from blood samples. Data shows that diabetic oral complex patients showed high RBS, HbA1c, FBS, and PBSS. Thus, dental condition is linked to diabetes.

## Background:

Diabetes mellitus is a disease which is because by a defect in insulin secretion and increased blood glucose level [1]. It has become a global epidemic and its complications impact the quality of life of people. According to World Health Organization, 171 million people had diabetes in 2000 [2]. Diabetes has been classified into 4 main types: Type 1, type 2, gestational diabetes and specific types of diabetes due to other causes. Type 1 and 2 are associated with the development of micro and macrovascular complications and these include retinopathy, nephropathy, and peripheral artery disease there is also an increased risk of diabetic foot and application [3]. Diabetes mellitus damages many organs like the heart, blood vessels, kidneys, eyes and nerves. People with diabetes are more likely to have mouth problems. It leads to problems like burning in the mouth, tooth decay, and so on. There are two ways in which diabetes and gum disease affect each other. Periodontitis and gum inflammation are more likely to happen in people with diabetes. Periodontal disease and type 2 diabetes have been linked for a long time. Controlling hyperglycemia is important because people whose diabetes is under control are less likely to get gum disease [4, 5]. Diabetes Mellitus also causes xerostomia. Diabetic patients will have salivary dysfunction, which will lead to decreased salivary flow and change in salivary composition. Salivary dysfunction is more common in diabetic patients. Diabetic patients are more susceptible to the development of new and recurrent dental caries. Chronic hyperglycemia causes irreversible pulpitis which will lead to pulp necrosis [6, 7, 8, 9].Burning sensation or dysesthesia in the oral cavity of diabetic patients is attributed to poor glycemic control, metabolic alterations in oral mucosa, candida infections [10]. Apart from this, our team has extensive knowledge and research experience that has translated into high-quality publications [10, 11, 12, 13]. Diabetes delays wound healing. Delaying or delayed healing of soft and hard tissue in diabetic patients will give complications during oral surgeries. Nonetheless, people with diabetes are mostly unaware of the association between diabetes and oral health and of their increased risk of various oral health complications. Therefore, it is of interest to evaluate diabetes associated with oral complications in the South Indian cohort [14].

##  Methodology:

The sample was collected in the Saveetha dental college and hospitals after obtaining institutional ethical approval with the number (SRB/SDC/UG-2118/22/495 on 21st June 2022). During the period of late July to September 2022, we collected around 20 samples for the study of diabetes-associated oral complications. In that 10 samples are non-diabetic control and test- 10 samples (diabetic + oral complications).

## Inclusion criteria:

Patient with oral complication without diabetes and with diabetes

## Exclusion criteria:

Patient with diabetes-associated comorbidities

The blood sample is collected in two tubes (1) Clot tube for blood sugar and (2) An EDTA tube for HbA1C.

[1] The 2 ml blood sample is collected in the clot tube (in red colour), and the serum is isolated with the centrifuge at 2000rpm for 8 min. The isolated serum is used for the determination of fasting blood sugar, postprandial blood sugar and random blood sugar.

[2] The 2ml blood sample is collected in an EDTA tube (purple colour), and the whole blood is used for the determination of the HbA1C.

This sugar level and HbA1C level is identified using a fully automated biochemistry analyzer (A15 biosystem).

## Statistical significance:

The statistics were done using IBM Statistical Package for the Social Science (SPSS) statistics 23 (t-test and one-way ANOVA have been performed).

##  Results:

**Figure 1:**
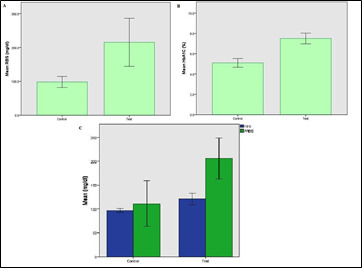
(A) Random blood glucose level of the control and oral complication in diabetes patients, (B) HbA1C of the control (5.10 ±0.45) and oral complication in diabetes patient, (C) the FBS and PPBS of the control and oral complication in diabetes patients

**Figure 2:**
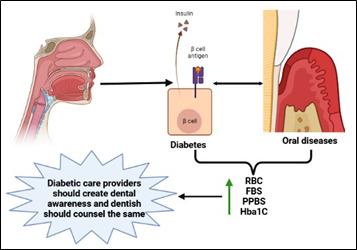
This figure describes the connection between diabetes and oral complication via altering various biochemical parameters.

The present study included 20 patients, 10 controls (oral complications without diabetes) and 10 patients (with diabetic with oral complications). Figure 1 shows the random blood glucose level of the control (102.60 ± 12.46) and oral complication in diabetes patients (204.16 ± 17.66). Figure 2 shows the HbA1C of the control (5.10 ±0.45) and oral complication in diabetes patients (7.42 ± 0.98). Figure 2 shows the FBS and PPBS of the control (96.75 ± 2.63 and 111.50 ±30.11) and oral complications in diabetes patients (121.40 ± 9.76 and 206.00 ± 34.53).

## Discussion:

The biochemistry parameters have been checked in oral complicated patients. Firstly we have reviewed the Random blood sugar level. The patients had an RBS level more than normal which is 140 mg/dL (Figure 1A). Next, we checked the hemoglobin level (HbA1C) of the oral health complicated patients. We found that the level of HbA1c was also higher than normal which is below 5.1 (Figure 1B). The post-prandial blood sugar and Fasting blood sugar was checked, Both the PPBS, and FBS in the oral complicated patients. So the oral complicated patients have more RBS, HbA1c, and PPBS significantly (Figure 1C). This indicates that they have diabetes. Most of the patients were not aware of this complication. The oral complications these patients came up with might be due to diabetes (Figure 2). So there should be enough awareness about the oral complications of diabetes. The difference in awareness about oral and systemic complications may be linked to high mortality and morbidity associated with systemic complications of diabetes [15]. At the start of the disease, patients with diabetes are not told that they are more likely to have complications. People with a low level of education don't know that diabetes is linked to other diseases because they aren't literate enough to learn about it on their own. In one study, it is shown that the patients do not know about their oral and health problems. Dentists should be on the lookout for signs and symptoms of diabetes that are both general and in the mouth [16, 17]. Patients with warning signs or abnormal blood glucose levels found by screening tests should be sent to a doctor for a diagnosis and any necessary treatment. Dentists should talk to their diabetic patients about how important it is to take care of their teeth and gums. Healthcare providers need to do more research to find out if they are giving the right advice to people with diabetes.

## Conclusion:

South Indian diabetics are unaware of their dental health risks. Therefore, diabetics and oral health patients must get personalized education about their elevated risk of complications. Hence, diabetic patients should prioritize oral health. Increased collaboration between oral and medical providers will be helpful in this context.
